# Effect of SP600125 on the mitotic spindle in HeLa Cells, leading to mitotic arrest, endoreduplication and apoptosis

**DOI:** 10.1186/s13039-016-0296-y

**Published:** 2016-11-25

**Authors:** Donia Mili, Kaouthar Abid, Imed Rjiba, Abderraouf Kenani

**Affiliations:** UR 12ES08 “Signalisation Cellulaire et Pathologies” Faculté de Médecine Monastir, Université de Monastir, Monastir, Tunisie

**Keywords:** SP600125, HeLa cells, Mitotic spindle, Apoptosis

## Abstract

**Background:**

The JNK inhibitor SP600125 strongly inhibits cell proliferation in many human cancer cells by blocking mitosis progression and inducing cell death. Despite, all this study, the mechanism by which SP600125 inhibits mitosis-related effects in human cervical cells (HeLa cells) remains unclear. In this study, we investigated the effects of SP600125 on the cell viability, cell cycle, and on the spindle assembly during mitosis in HeLa cells.

**Methods:**

To explore this approach, we used a viability test, an immunofluorescence microscopy to detect Histone phosphorylation and mitotic spindle aberrations. Apoptosis was characterised using Western Blotting.

**Results:**

Treatment of HeLa cells with varying concentrations of SP600125 induces significant G2/M cell cycle arrest with elevated phosphorylation of histone H3 within 48 h, and endoreduplication after 48 h. SP600125 also induces significant abnormal mitotic spindle. High concentrations of SP600125 (20 μM) induce disturbing microtubule assembly in vitro. Additionally, SP600125- induced delayed apoptosis and cell death was accompanied by significant poly ADP-ribose polymerase (PARP) cleavage and caspase-3 activation in the late phase (at 72 h).

**Conclusion:**

Our results confirmed that SP600125 induce mitosis arrest in G2/M, endoreduplication, mitotic spindle aberrations and apoptosis.

## Background

Faithful transmission of genetic information during mitosis is ensured by the spindle assembly checkpoints [1]. Cell cycle progression to the G1, S, and G2/M phases is controlled by those cell cycle checkpoints that ensure the correct order and transition timing of the mitotic spindle [2]. After G2/M arrest, a significant subpopulation of pRb-negative cells demonstrated an excessive amount of 4 N DNA, known as endoreduplication [3]. Many agents are known for their effect of endoreduplication: agents that interfere with spindle assembly (eg. Microtubule polylerisation inhibitors eg., colchicines), the enzyme poisons (eg: amsacrine, and Adriamycin), catalytic inhibitors (eg: merbarone, aclarubicin) and physical agents that damage DNA, such as X-rays. Some of these microtubule-interfering agents, such as nocodazole and paclitaxel, induce significant endoreduplication due to the sister chromatid miss-segregation [4].

SP600125 is an anthrapyrazolone inhibitor of JNK that competes with ATP to inhibit the phosphorylation of c-Jun. Although JNK appears to be involved in cell proliferation, there is no evidence linking JNK activation to specific phases of the cell cycle. In fact, in Jurkat cells, JNK activity increased in G2/M checkpoint and was demonstrated to be responsible for apoptotic Bcl-2 phosphorylation [5]. Recent studies have focused on the effects of JNK in the promotion of cell death, and it has been reported that the JNK-antisense oligonucleotide inhibits tumour growth and induces regression in a high number of cases [6, 7].

Some research indicates that JNK activity is essential to maintain proliferation and diploidy in cancer cells. However, the induction mechanisms of G2/M arrest, endoreduplication, and apoptosis due to SP600125 still unclear [8].

In this study, we investigated the relationships between polyploid giant cells and apoptosis in human cervical cells treated with SP600125. We tried to explore the effects of SP600125 in G2/M phase arrest, endoreduplication, and delayed apoptosis via disorganization of the mitotic spindle assembly.

## Methods

### Reagents

The specific JNK inhibitor SP600125 was purchased from Sigma (S5567). The inhibitor was reconstituted in DMSO to make a 10 mM stock solution, and DMSO (0.1%) was used as a control vehicle. MTT, Hoechst 33258. (861405 Sigma Aldrich), were purchased from Sigma (St. Louis, MO). Triton and FBS were purchased from GIBCO (Gaithersburg, MD). An enhanced chemiluminescence (ECL) kit was purchased from Amersham (Arlington Heights, IL). Rabbit Anti-Histone H3 (phospho S10) antibody (ab47297). Primary antibodies were detected using an anti-mouse and anti-rabbit secondary antibodies conjugated with Alexa Fluorescent.

### Cell lines and cell culture

HeLa human carcinoma epithelial cell lines were obtained from the American type culture collection (ATCC. CCL-2). Cells were grown in DMEM supplemented with 10% heat-inactivated FBS and 1% penicillin- streptomycin (GIBCO) in 5% CO2 at 37 °C. Cells were seeded at 5 × 10^4^ cells/ml and treated with SP600125 at the indicated times. Cell growth was determined using MTT assays.

### Immunofluorescence analysis

For immunofluorescence analysis, Firstly, we brought the cells, already cultured and transfected on a glass coverslip in a 24-well plate. The cells were washed three times with phosphate-buffered saline (PBS) and then fixed with PBS containing 4% paraformaldehyde at room temperature for 30 min. The fixed cells were washed with PBS containing 0.1% Triton X-100 and then blocked with 1 mg/ml NaBH4 in PBS. After been blocked, the cells were incubated for 1 h with the primary antibodies, mouse monoclonal anti-α-tubulin (F2168 clone DM1A purified immunoglobulin, monoclonal anti-α-tubulin-FITC antibody produced in mouse, Sigma Aldrich).

The cells were then washed three times with PBS containing 0.1% Triton X-100 and incubated with a secondary antibody, Alexa-fluor 488 anti- Mouse antibody (62197 Sigma Aldrich) and Alaxa-fluor 568 anti-rabbit Antibody (SAB1102713 Sigma Aldrich) for 20 min, at room temperature and away from light.

For DNA staining, cells were incubated with 5 μg/ml Hoechst 33342 for 5 min prior to fixation. Glass coverslip was mounted on a glass slide with the Fluor save (Calbiochem 34 5789) and examined under a fluorescence microscope.

### Statistical analysis

All data from cell counts, MTT assays, PARP and caspase-3 activity experiments were derived from at least three independent experiments. Images were visualized with a Zeiss Axiovert 200 M inverted microscope. Images were captured using CoolSnap HQ (Photometrics) black and white camera driven by the Metamorph software (Universal Imaging) then downloaded into Photoshop. Image J (1.49 V) was used to quantify the Western blots. Statistical analyses were conducted using Sigma Plot software, and values are presented as mean ± SD. Significant differences between groups were determined using an unpaired Student’s t-test. A value of *P < 0.05 was accepted as an indication of statistical significance.

## Results

### SP600125 induces a dose-dependent inhibition in cell viability and expansion of cell size in human HeLa cells

**Fig. 1:**
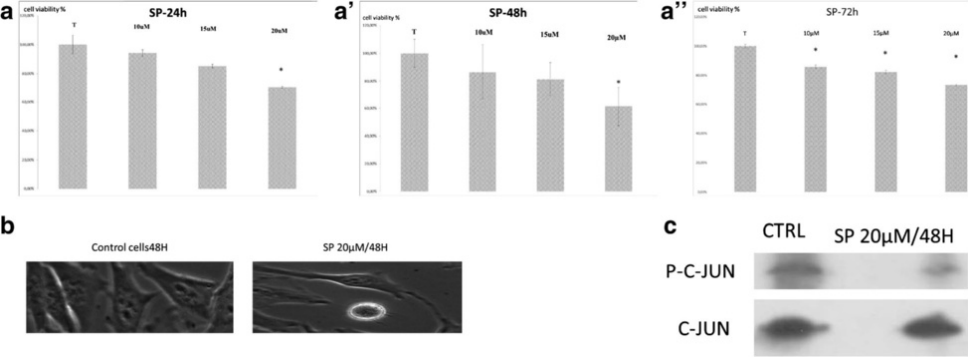
**a** HeLa cells were plated at 5 × 104 cells/ml and incubated for 24 h. Cells were treated with the indicated concentrations of SP600125 and the cell viability was measured using a metabolic-dye-based MTT assay. Each point represents the mean ± SD of three independent experiments. Significance was determined using Student’s test (*P < 0.05 vs. vehicle control). **a**’ HeLa cells were plated at 5 × 104 cells/ml and incubated for 48 h. Cells were treated with the indicated concentrations of SP600125 and the cell viability was measured using a metabolic-dye-based MTT assay. Each point represents the mean ± SD of three independent experiments. Significance was determined using Student’s test (*P < 0.05 vs. vehicle control). **a**”: HeLa cells were plated at 5 × 104 cells/ml and incubated for 72 h. Cells were treated with the indicated concentrations of SP600125 and the cell viability was measured using a metabolic-dye-based MTT assay. Each point represents the mean ± SD of three independent experiments. Significance was determined using Student’s test (*P < 0.05 vs. vehicle control). **b** Cells were incubated with the indicated concentrations of SP600125. After a 48 h-incubation, cells were sampled and examined under light microscopy. Magnification × 200. **c** After HeLa cells were treated with SP600125 for the indicated time, expression of c-Jun and phosphorylated c-Jun was detected using Western blot analysis

Recent studies have shown that SP600125, a pharmacological inhibitor of JNK, causes cell viability inhibition in certain cell types, including breast cancer [8], multiple myeloma [9], and B-lymphoma [10]. To verify this effect on cervical cells, HeLa cell line was treated with varying concentrations of SP600125 for 24 h, 48 h and 72 h. Cell viability and morphological changes were assessed using MTT assays and microscopy (Fig. 1).

As shown in Fig. 1(a, a’, a”) significant inhibition of cell viability in a dose/time-dependent manner was observed in HeLa cell line. DMSO (0.1%), used as a vehicle control, did not affect cell viability or morphology.

In other hand, when cells were examined under phase contrast microscopy, after 48H of incubation cells treated with 20 μM SP600125 presented with swelling, condensed nucleus and modest apoptotic shrinkage at 48 h, compared to control cells (Fig. 1b). To confirm that SP600125 inhibited JNK activity, we realised a Western blot analysis using p-c-Jun antibodies in HeLa cells extract after their incubation with SP600125 during 48H. As shown in Fig. 1c, treatment with 20 μM SP600125 almost completely supressed c-Jun phosphorylation after 48 h. These results indicate that SP600125 causes anti-proliferative effects with an apoptotic cell morphology in cervical cells through inhibition of JNK activity.

### SP600125 treatment induces persistence of histone H3 phosphorylation in HeLa cells during mitosis

Some research has shown that Serine 10 (Ser^10^) phosphorylation of histone H3 has emerged as a sensitive marker for mitotic cells [11]. In mammalian cells, site-specific phosphorylation of H3 at Ser^10^ has been shown to initiate bulk phosphorylation during prophase, become maximal during metaphase, diminish during anaphase and is lost during telophase. Drugs that induce the phosphorylation of H3 also initiate premature chromosomal condensation in cell lines. Also, drug-induced dephosphorylation of H3 were correlated with chromosome relaxation [12].

**Fig. 2:**
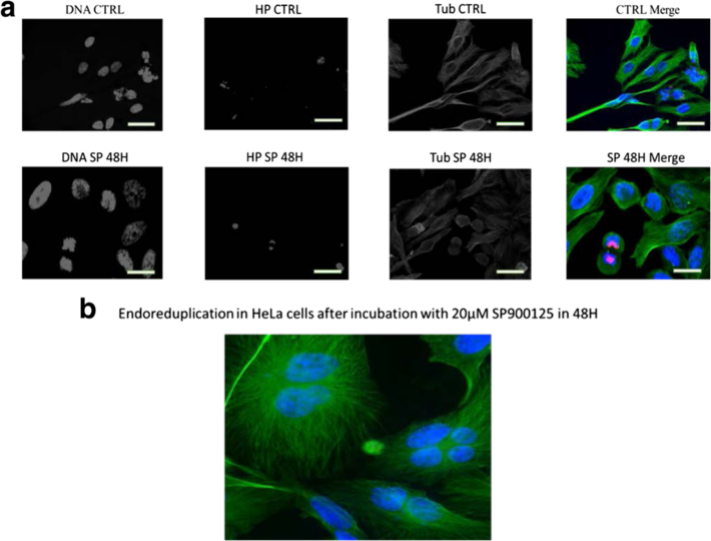
**a** Histone 3 Phosphorylation was analyzed using immunofluorescent staining in HeLa cells treated with 20 μM SP600125 for the indicated times. Cells were fixed, permeabilized, and stained. Tubulin tagged with IgG anti-tubulin, H3 phosphorylated was stained with Rabbit Anti-Histone H3 (phospho S10) antibody (ab47297). Primary antibodies was detected using an anti-mouse and anti- rabbit secondary antibodies conjugated with Alexa Fluorescent (*Green* for tubulin and *Red* for H3P). Cells were analyzed using fluorescence microscopy (×400). Hoechst 33258 was used for nuclear staining. **b** Endoreduplication detected using Immunofluorescence in HeLa cells treated with 20 μM SP600125 for 48H. Tubulin tagged with IgG anti-tubulin. Primary antibody was detected using an anti- mouse secondary antibody conjugated with Alexa Fluorescent (*Green* for tubulin)

In this study, we tried to use this approach by using antibodies highly selective to the amino-terminus of H3 in a phosphorylated Ser^10^ form. As shown in Fig. 2a, Ser^10^ phosphorylation of histone H3 was retained in HeLa cells at 48 h. SP600125 time-specifically induced G2/M phase arrest at 48 h with histone H3 phosphorylation on Ser^10^ as a G2/M arrest marker, and then induced endoreduplication at 48 h (Fig. 2b). Finaly HeLa cells presented significant signs of apoptosis and endoreduplication.

### SP600125 induces the formation of aberrant mitotic spindle

Microtubules (MTs) play an important role in cell replication and division, maintenance of cell shape, and cellular movement. Microtubules are composed of α-, β-tubulin, and microtubule-associated proteins (MAPs). They are in an unstable steady state of a highly dynamic process of polymerization and depolymerization, and disrupting the dynamics of microtubules leads to endoreduplication.

**Fig. 3:**
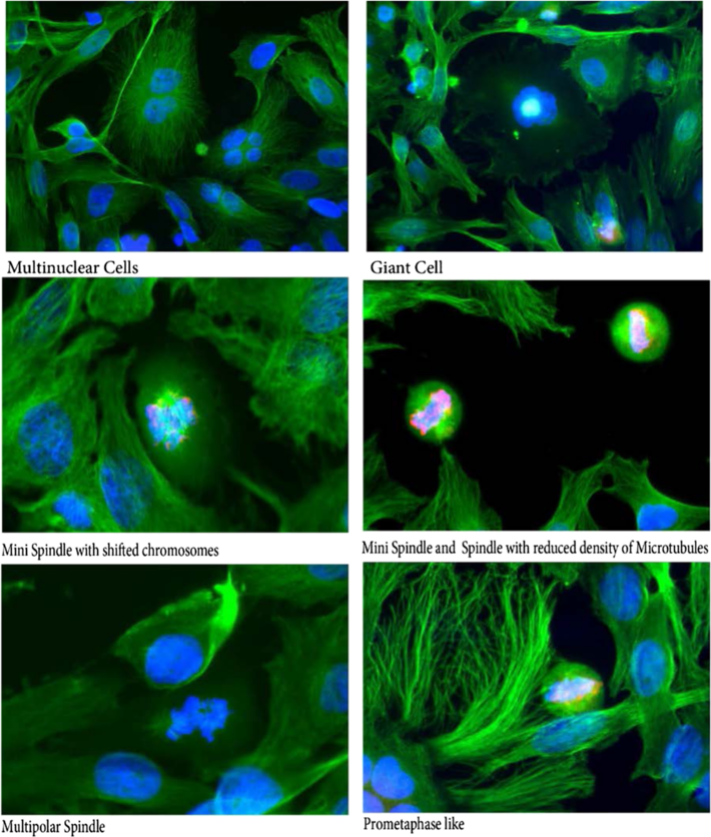
Immunofluorescence analysis shows clearly the formation of aberrant mitotic spindle structure. Spindle perturbation in HeLa cells after SP600125, Tubulin tagged with IgG anti-tubulin (*green* fluorescence), and DNA labelled with Hoechst

In order to examine the MTs assembly in SP600125- mediated endoreduplication, we reasoned that the mitotic spindle itself might be a target of SP600125 effect. To respond to this question, we examined whether mitotic cells treated with 20 μM SP600125 displayed many changes in tubulin polymerization. Treatment with SP600125 increased the abnormal structure and promoted an increased intensity of α-tubulin staining, measured by indirect immunofluorescence (Fig. 3). Immunofluorescence analysis does not clearly provide a quantitative measure of tubulin polymerization in the cell but it shows the formation of aberrant structures: mini spindle with shifted chromosomes, mini spindle and spindle with reduced density of microtubules, multipolar spindle as shown in Fig. 3 [13].

### SP600125 induces apoptosis in HeLa cells after endoreduplication and aneuploidy

To assess whether delayed apoptosis contributed to the viability inhibition effects of SP600125, we investigated the effects of SP600125 on apoptosis.

Apoptosis is controlled by a complex interplay between many proteins. Bcl-2, a 26-kDa integral membrane oncoprotein, was the first anti-apoptosis gene product discovered. Several research has demonstrated that overexpression of Bcl-2 protein protects cells from apoptosis in some cell lines [12]; although a recent report indicates that the level of this oncoprotein is not always correlated with an increased ability of the cell to resist death-promoting stimuli [14]. Recently, some research, reported that treatment with either okadaic acid, a potent inhibitor of phosphatase, or the antitubulin agent paclitaxel induced in Bcl-2 protein phosphorylation and induction of programmed cell death in lymphoid cells. Suggesting that Bcl-2 phosphorylation may change its antiapoptotic function. Whereas anticancer drugs damaging DNA do not [12].

**Fig. 4:**
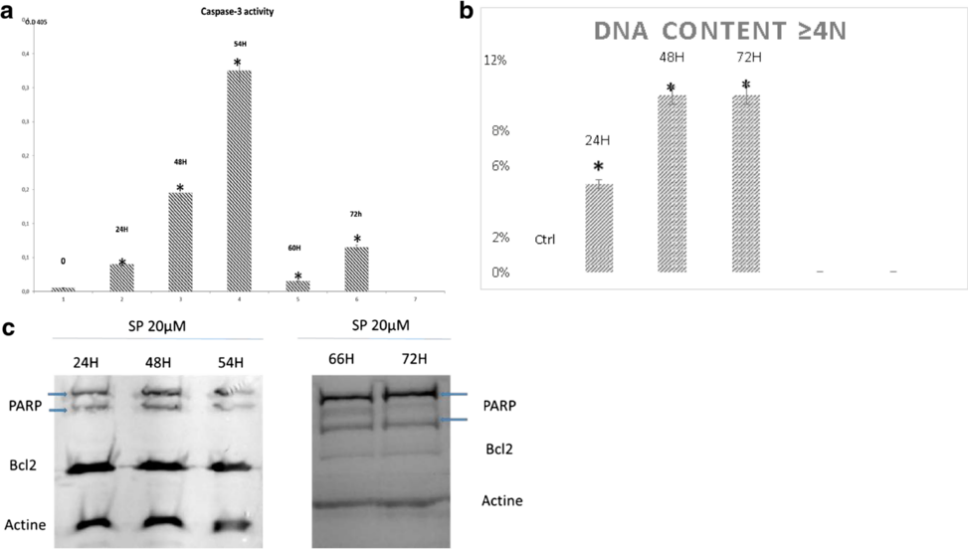
**a** SP 20 μM, after 48 h of treatment with SP600125, the caspase-3 activity was assayed using a caspase assay kit, following the manufacturer’s protocol. **b** Quantification of Multinucleated cells observed because of the SP600125 incubation (20 μM). Time dependent experience. The average value ± SD from three independent experiments is also shown. Asterisks indicate significant differences (* *p* < 0.05) calculated by the Duncan’s t-test. **c** Equal amounts of cell lysate (60 μg) were resolved using SDS-PAGE, transferred to nitrocellulose, and probed with specific antibodies (anti-PARP and anti-Bcl-2). Actin was used as an internal loading control

In this study, in HeLa cells, SP600125 (20 μM) induced an increase in the multinuclear giant cell population (>4 N DNA) (Fig. 4b) and the caspase-3 activation (Fig. 4a) in a time-dependent manner. Western blot analysis also demonstrated that SP600125 caused PARP cleavage and Bcl-2 downregulation (Fig. 4c), suggesting that the inhibitory effects of SP600125 on cervical cell viability are dependent on apoptosis.

## Discussion

SP600125 has been implicated in G2/M arrest and apoptosis, but its precise role remains unknown [15]. The present study provides the mechanism to explain the induction of G2/M arrest, endoreduplication, and delayed apoptosis caused by SP600125 in cervical cells.

As shown in Fig. 2a, we have demonstrated that SP600125 (I- arrests G2/M phases with phosphorylation of histone H3 at 24 h; (II- suggesting that SP600125 induces endoreduplication; (III- promotes spindle aberrations, a critical process in cell division; and (IV- induces delayed apoptosis in HeLa cells. Therefore, SP600125 has a strong anticancer effect against cervical cells in a dose- and time-dependent manner by disturbing tubulin polymerization and disrupting the organization of the microtubule mitotic cytoskeleton.

The G2/M checkpoint is especially important in protecting normal cells from tumour formation driven by the accumulation of mutations [16]. Therefore, elimination of the checkpoint increases the sensitivity of human tumour cell lines to anticancer agents. Some studies have reported that the G2/M arrest induced by SP600125 may be due to inhibition of cyclin B/Cdk1 kinase activity through an increase in p21 levels [17, 18]. Increased JNK activity is important for the dissociation of p21 and JNK, following which cells enter into the S phases [8].

Using biochemical and immunofluorescence methods, we have shown that SP60015 significantly increases tubulin disorders. Microtubules are crucial cellular and structural components that induce cellular development, division, and movement [19]. Therefore, microtubule-disrupting agents provide a novel approach to cancer chemoprevention and/or cancer therapy.

Recently, certain cancer chemotherapy agents have been found to exert their anticancer activities by disrupting the dynamics of microtubule assembly, thus perturbing the formation and function of the mitotic spindle apparatus and arresting cells in mitosis [20, 21]. This action of SP600125 is similar to that of paclitaxel, which binds to tubulin and increases tubulin polymerization, causing cells to arrest in the G2/M phase thereby blocking cell cycle progression [13]. Our results strongly support the idea that SP600125 inhibits cell proliferation by inhibiting mitosis through disturbing tubulin polymerization.

Tumour cells often evade apoptosis by overexpressing anti-apoptotic proteins, such as Bcl-2, which give them a survival advantage [22]. Recently, contrasting results have been reported. In fact, decreased or phosphorylated Bcl-2 is implicated in the resistance of human ovarian cancer cells to tubulin polymerizing agents, such as paclitaxel [23, 24]. Other reports have shown that Bcl-2 phosphorylation is a common event in mitosis [14].

Our results have shown that the level of endogenous Bcl-2 expression does not affect SP600125-induced endoreduplication up to 24 h (Fig. 4c).

## Conclusion

In conclusion, our findings indicate a role for both targeting (tubulin polymerization) and signalling (Bcl-2) in human cervical cells for SP600125. Increased p-histone H3 protein expressions were found to be responsible for SP600125-induced G2/M arrest at 48 h and high SP600125-induced endoreduplication at 48 h. SP600125-induced delayed apoptosis was related to Bcl-2 expression, which was closely related to endoreduplication. Further studies are necessary to clarify the exact mechanisms that are induced by SP600125 involved in specific stages of cell apoptosis.

## Abbreviations

ATP: Adenosine triphosphate
DNA: Deoxyribonucleic acid
H3: Histone 3
JNK: Jun kinase
MAPs: Microtubules associated proteins
MTs: Microtubules
PARP: Poly ADP-ribose polymerase (PARP)
Ser: Serine
